# Characterization of spatial distribution of the bacterial community in the South Sea of Korea

**DOI:** 10.1371/journal.pone.0174159

**Published:** 2017-03-17

**Authors:** Ji-Hui Seo, Ilnam Kang, Seung-Jo Yang, Jang-Cheon Cho

**Affiliations:** Department of Biological Sciences, Inha University, Incheon, Republic of Korea; Universidad Autonoma Metropolitana, MEXICO

## Abstract

In order to investigate the importance of spatial and environmental factors on the structure and diversity of bacterial communities, high-resolution 16S rRNA gene tag pyrosequencing was applied to bacterial communities in the littoral sea. Seawater samples were prepared from seven different stations in the South Sea of Korea, the marginal sea in the western Pacific Ocean, and were divided into three groups according to distances from the coastline. The majority of 19,860 sequences were affiliated with *Alphaproteobacteria* (58.2%), *Gammaproteobacteria* (7.9%), and *Bacteroidetes* (13.9%). The bacterioplankton community at each station was highly diverse and varied among the samples. Major bacterial lineages showed different niche preferences among three locational groups. *Alphaproteobacteria* was the most abundant bacterial class, and it harbored the most frequently recorded operational taxonomic units (OTUs) in all sampling stations. However, dominant groups at the order levels showed a clear difference among the samples. The SAR11 clade was more abundant in coastal waters while the *Roseobacter* clade prevailed at stations far away from the coastline. Furthermore, members of *Actinobacteria* and *Cyanobacteria* also exhibited spatial variability. The OM1 clade in *Actinobacteria* constituted a predominant fraction in coastal samples, but it was essentially absent at the distal stations closer to open ocean. In contrast, *Synechococcus* was the predominant taxon in the distal samples, accounting for 7.1–19.5%, but was hardly detected in coastal waters, representing less than 0.7%. In *Bacteroidetes*, NS5 and NS9 groups tended to inhabit coastal waters while the genera *Polaribacter* and *Ulvibacter* were more abundant in distal stations. Clustering analysis and principle coordinates analysis based on OTU data indicated that bacterial communities in the studied area were separated into three groups that coincided with locational grouping. Statistical analysis showed that phosphate and dissolved oxygen concentration had a significant influence on the bacterial community composition.

## Introduction

Microorganisms are the most abundant and successful organisms on earth, but relatively little is known about the biogeographic distribution patterns of microbial communities within and between major habitats. Microbial biogeographical studies describe spatiotemporal distribution patterns of microbes and elucidate common aspects of the correlations between community composition and characteristics in different environments [1]. Spatiotemporal patterns in microbial community structure are well structured and highly influenced by geographical characteristics and environmental variables [2, 3]. Microbial biogeography can be summarized by the historic phrase “everything is everywhere, but the environment selects.” It implies that despite no limitation on bacterial dispersal in the environment, there is niche differentiation among microbial taxa selected as competitive survivors adapted to distinct environmental conditions. To explain niche-based relationships between major bacterial populations and environmental variation, many studies identified a number of different environmental parameters, such as NH_4_^+^ and total organic nitrogen concentration [4], phosphate concentration [5], temperature [5, 6], and salinity [7, 8], affecting the dynamics of bacterioplankton communities.

Recent advances in molecular phylogenetic approaches have revolutionized our view of microbial diversity and provided enhanced understanding of the complexity of microbial communities in marine environments. Furthermore, these studies have revealed that some microbial taxa exhibit distinct seasonal and biogeographical distribution patterns in the ocean, along with varying environmental parameters [9–11]. Since physicochemical and biological differences in a coast-to-ocean transect are profound, this environmental heterogeneity has been considered a very powerful factor acting on microbial populations. For example, the SAR11 and *Roseobacter* clades showed opposite peaks in seasonal abundances and represented different distribution patterns along with marine geographic regime [4, 9]. SAR11 bacteria tend to prevail in the winter season, while members of the *Roseobacter* clade are known to be widespread in spring and autumn.

Studies on the variation in microbial communities in the ocean have helped us to understand the long-term responses of marine bacterioplankton to environmental changes [12] and that bacterioplankton plays key roles in marine biological processes [13]. However, even with improved molecular tools, it is not easy to determine the extent to which interactions among environmental factors govern ecosystems and biogeochemical processes. Because of the contiguous habitats in the ocean, it is difficult to set habitat boundaries and distinguish the influences of environmental factors on the diversity of bacterioplankton. Thus, answering this question requires information on the microbial diversity patterns and the spatial arrangement of the sampled assemblages.

The South Sea of Korea is a marginal sea located in the western Pacific Ocean, an area characterized by a group of islands, an archipelago. Thus, the South Sea of Korea is influenced by a variety of different oceanographic processes, including freshwater discharge from islands and rivers and the interaction of different currents. The highly dynamic and complex hydrological condition in this sea affects the composition of bacteria communities along the nutrient gradient from coast to open oceanic region. Therefore, these geographical positions might be suitable to explain correlations between bacterial community composition and environmental factors along with the spatial variations in this region and thus, contribute to our understanding of the general distribution bias of bacterioplankton in the ocean.

In this study, bacterial communities of seawater masses characterized by varying distances from the coastline were assessed by applying the 454-pyrosequencing method and physicochemical characteristics such as phosphate concentration that greatly impact bacterioplankton diversity were also analyzed. The bacterioplankton community in the South Sea of Korea showed spatial variations along transects from the coast to the oceanic zone. Distinct bacterial groups prevailed in different water masses and this pattern was correlated with environmental parameters.

## Materials and methods

### Ethics statement

No specific permits were required for the described field studies. The location is not privately owned or protected in any way, and the field studies did not involve endangered or protected species.

### Sample collection and analyses of environmental parameters

**Fig 1:**
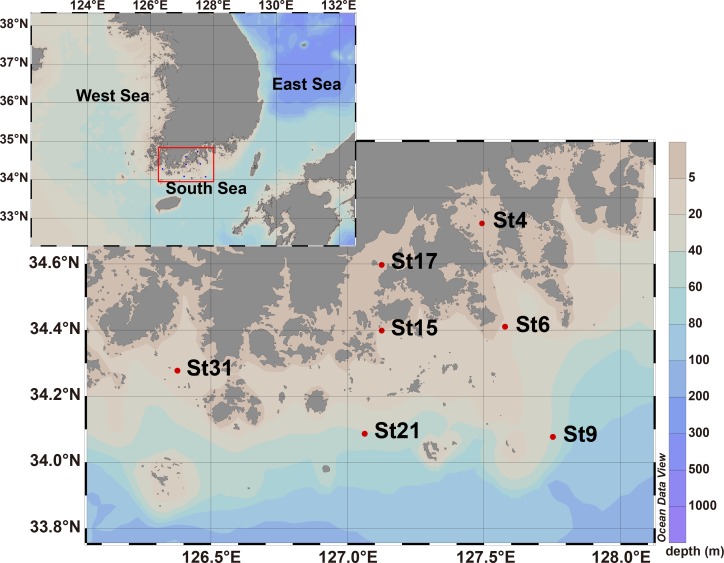
Map showing the sampling stations in the South Sea of Korea. Samples of St4, St6, St9, St15, St17, St21, and St31 were collected from surface seawater in May 2009.

Seawater samples were collected from the surface at 7 different stations along the coastline and transects from the coastal to distal region in the South Sea of Korea in May 2009 (Fig 1). The sampling stations were selected along distance from the coastline: St4 (13.52 km), St6 (49.05 km), St9 (88.95 km), St15 (33.83 km), St17 (11.80 km), St21 (68.84 km), and St31 (31.24 km). The water samples were collected using Niskin bottles attached to a conductivity, temperature, and depth (CTD) rosette multi-sampler. Temperature and salinity profiles were obtained on the downward casts of the CTD rosette profiler (Seabird-911). Dissolved oxygen (DO) was quantified using the Winkler method and pH was measured using a pH meter (Mettler). Total suspended solids (TSS) were measured by filtering the seawater through a pre-weighted GF/F filter (Whatman). Nitrate (NO_3_^−^), nitrite (NO_2_^−^), ammonium (NH_4_^+^), phosphate (PO_4_^3−^), and silicate (SiO_3_^2−^) concentrations were quantified using a Flow Injection Analyzer (Lachat instruments, CO, USA). The concentration of chlorophyll *a* (Chl *a*) was measured in 90% acetone extracts using a fluorometer and finally estimated in accordance with the equation of Humphrey and Jeffrey [14]. All data for these environmental parameters are available from open research data source on marine physicochemical studies (http://webgis.ecosea.go.kr/). A summary of the physiochemical characteristics of the sampling stations is shown in Table 1. Microbial biomass was collected from seawater samples and concentrated by filtration on the research vessel. One liter of each sample was filtered directly through 0.2-μm pore size, 47-mm-diameter polyethersulfone Supor membrane filters (Pall Corp., NY, USA) and the membrane filters were then stored at −80°C until use.

**Table 1:** Environmental parameters in the sampling stations.

Station	Latitude	Longitude	Temperature (°C)	Salinity (psu)	pH	DO (mg/L)	TSS (mg/L)	PO_4_^3−^ (μM)	DIN^*^ (μM)	Silicate (μM)	Chl *a* (μg/L)
**St4**	34°43′22.8″N	127°29′34.8″E	19.98	33.62	8.05	8.12	3.31	0.26	1.29	25.32	2.69
**St6**	34°24′36.0″N	127°34′37.2″E	15.97	33.62	8.13	8.50	1.60	0.22	4.25	5.55	3.58
**St9**	34°04′37.2″N	127°45′07.2″E	16.15	33.57	8.21	9.39	0.40	0.02	1.23	1.08	0.56
**St15**	34°23′56.4″N	127°07′26.4″E	15.76	33.33	8.16	8.71	3.00	0.19	2.62	3.41	1.12
**St17**	34°35′49.2″N	127°07′26.4″E	17.71	32.87	8.06	7.84	1.93	0.18	1.88	8.22	1.07
**St21**	34°05′13.2″N	127°03′46.8″E	14.56	33.50	8.12	8.80	0.52	0.10	2.31	4.64	1.25
**St31**	34°16′37.2″N	126°22′40.8″E	14.43	33.32	8.15	8.63	12.43	0.19	3.46	3.93	1.81

DO, dissolved oxygen; TSS, total suspended solids; DIN, dissolved inorganic nitrogen; Chl *a*, chlorophyll *a*

* Sum of nitrate, nitrite, and ammonium concentration

### DNA extraction and Polymerase Chain Reaction (PCR) amplification for pyrosequencing

DNA was extracted from the membrane filters by enzymatic lysis using lysozyme, proteinase K, and sodium dodecyl sulfate [15]. The extracted DNA was further purified using the kit (GeneAll Biotech., Korea) and then stored at −20°C until use for polymerase chain reaction (PCR) amplification. The V1-V3 hypervariable regions of the bacterial 16S rRNA genes were amplified for 20 cycles using fusion primers that were designed based on universal primers, 27F (5′-GAGTTTGATCMTGGCTCAG-3′) [16] and 519R (5′-WTTACCGCGGCTGCTGG-3′) [17] with the addition of unique six- to eight-nucleotide barcode sequences and the 454 FLX-titanium adaptor sequences. Pyrosequencing of pooled PCR products was performed using a Roche 45h4 GS FLX Titanium platform at ChunLab, Inc. (Seoul, Korea).

### Pyrosequencing data processing and analysis

The open-source, platform-independent, community-supported software program, Mothur (Mothur v. 1.36.1; http://www.mothur.org) was used to process and analyze the raw pyrosequencing data [18]. The pyrosequencing reads were processed by removing barcodes and primers and only accepting reads with an average quality score above 25 and read lengths longer than 300 nucleotides. Data analysis was carried out following the Schloss standard operating procedure [18, 19]. Reads were aligned based on a SILVA reference alignment and chimeric sequences were removed using UCHIME [20]. Taxonomic assignment was performed using the SILVA databases (https://www.arb-silva.de/documentation/release-119/) at a 60% confidence threshold with the RDP classifier implemented in Mothur. After phylogenetic allocation of the sequences down to the phylum, class, order, and genus level, relative abundance of a given phylogenetic group was set as the number of sequences affiliated with that group divided by the total number of sequences per sample. Clustering of sequences into operational taxonomic units (OTUs) was performed using an average-linkage-based algorithm with a 98% similarity cutoff. A representative sequence from each OTU was selected after clustering analysis. Subsampling was performed to normalize the sequence number for analyses. This involved random selection of a number of sequences from each sample that corresponded to the smallest number of sequences from an individual sample.

### Statistical analysis

Statistical analyses were performed in R (v3.2.2, R Development Core Team, 2015), using packages *vegan* and *ade4* with normalized sequences. Population diversity (Shannon index), evenness (Shannon’s evenness index), and richness (ACE, Chao1) were calculated and compared between groups [21]. Shannon diversity index and species richness estimator Chao1 were generated using R for each sample. Bray-Curtis and Euclidean dissimilarity indices were used to generate community distance matrices and environmental distance matrices, respectively [22]. These dissimilarity matrices were then used to create a dendrogram, using weighted group average linkage in cluster analysis. To identify major environmental parameters influencing bacterial community structure, relative distances among samples were visualized in non-metric multidimensional scaling (NMDS) plots generated from Euclidean similarity index matrices of Chord-transformed OTU data by using the ‘metaMDS’ function and environmental parameters were fitted to the ordination plots using the ‘envfit’ function of the *vegan* package in R. To identify the indicator OTUs that characterized each of the seawater samples analyzed, the Indicator Species Analysis approach was applied in R, using package *labdsv* (http://ecology.msu.montana.edu/labdsv/R) and test *indval* [23]. The statistical significance of the resulting IndVal was evaluated for each species by a random re-allocation procedure of stations (999 permutations) among the three groups (Group A, Group B, Group C)—defined by cluster analysis. Only OTUs with IV > 0.4 and P < 0.05 were considered good indicators.

## Results and discussion

### Overview of pyrosequencing-generated bacterial diversity

Recent biogeographical studies have accumulated plenty of data on the abundance of bacterial populations in time and space scales [4, 24–26]. Current evidence supports that environmental factors are responsible for spatial variation in marine microbial diversity. In the current study, we investigated marine bacterial diversity in the regional sea of the marginal western Pacific Ocean, which is influenced by river runoff and nutrient input. To understand the dependence of marine bacterial community structure on distance from the coastline, 7 seawater samples were collected from the South Sea of Korea (Fig 1) and physicochemical parameters were determined (Table 1). Based on the locations from the coastline, 7 stations could be arbitrarily divided into 3 groups: St4 and St17 (10 ~ 20 km, Group A), St6, St15, and St31 (30 ~ 50 km, Group B), and St9 and St21 (60 ~ 90 km, Group C). Interestingly, these 3 arbitrary groups showed the same grouping pattern in the cluster analyses based on OTUs, which will be discussed later. Physicochemical parameters including temperature, salinity, pH, DO, TSS, phosphate, dissolved inorganic nitrogen (DIN), and chlorophyll *a* concentrations were measured to analyze the water condition of the South Sea of Korea. Water temperature in the studied area was in the range of 14.43 ~ 19.98°C, showing higher temperature in the coastal stations. DO concentration was in the range of 7.84 ~ 9.39, showing the highest concentrations in the distal stations (9.39 in St9 and 8.8 in St21). pH at the research stations was in the range of 8.05 ~ 8.21 and pH in the coastal area was lower than pH in the distal area. The concentration of phosphate was relatively higher at the coastal stations (St4, St6, and St17, 2.74 μM, 2.32 μM, and 2.00 μM, respectively) than at the distal stations (St9 and St21, 0.21 μM and 1.05 μM, respectively). The DIN concentration was slightly higher in the intermediate area (St6, 15, and 31; Group B) than stations of Group A and Group C. According to the assessment criteria proposed by Wasmund *et al*. [27], DIN and phosphate concentrations in the studied area were estimated to be oligotrophic in the distal stations and mesotrophic in the coastal stations. In a transect of St4, St6, and St9, chlorophyll *a* concentrations were much lower in St9 (0.56 μg/L) than those in St4 and St6 (2.69 μg/L and 3.58 μg/L, respectively). The gradient distribution of environmental parameters from the coastline to the open sea has been reported in other marine environments. In the South Atlantic and the North Atlantic Ocean, the concentration of phosphate was known to range between 0.8 μM and 1.7 μM in coastal stations and decreased to very low concentrations between 0.1 μM and 0.2 μM in oceanic waters [28–30]. The concentration of chlorophyll *a* was also reported to be high near the coast (> 2 μg/L) but low in the open ocean (< 0.5 μg/L) in the East China Sea [31]. The high concentrations of phosphate and chlorophyll *a* in coastal waters might be due to a load of nutrients from the land, and these gradually lessened further away from the coast. The concentration of total suspended solids that appeared high in the coastal area and low in the oceanic area also supported this gradual distribution of physicochemical parameters.

Using 16S rRNA gene pyrosequencing, a total of 34,034 raw pyrotag reads were obtained from 7 samples. After quality control filtering and chimera removing, 31,033 high-quality sequences were collected and they were clustered into 1,714 OTUs (S1 Table). After classification using the SILVA database classifier at a confidence threshold of 90%, 64% (19,860 sequences) of all qualified reads were assigned to the domain *Bacteria*. The remaining 36% of sequences were classified into the *Eukarya* (16S rRNA gene of chloroplasts and mitochondria) but these eukaryal sequences were out of this research scope and thus excluded from further analyses. The Shannon diversity index calculated from 19,860 bacterial sequences after OTU clustering showed that bacterial diversity was relatively higher in St6, St15, St21, and St31 than in St4, St9, and St17 (Table 2). Other diversity indices such as abundance-based coverage estimators (ACE), Chao1, and species evenness showed that there was no clear relationship between alpha-diversity and geographic sampling locations. Thus, in order to compare the bacterial diversity across the sampling stations, the abundance of bacterial taxa was investigated using phylogenetic analyses.

**Table 2:** Number of bacterial sequences, OTUs, and alpha-diversity indices.

	St4	St6	St9	St15	St17	St21	St31	Total
**No. of sequences**	4686	1974	5157	3134	2519	1438	952	19860
**No. of OTUs**	184	210	165	218	190	172	232	846^*^
**Ace**	441.19	514.72	479.87	624.85	736.00	520.18	796.67	−
**Chao1**	455.44	426.24	453.90	535.28	754.18	641.62	620.32	−
**Shannon**	3.31	3.81	3.32	3.95	3.21	3.76	3.51	−
**Evenness**	0.15	0.22	0.17	0.24	0.13	0.25	0.14	−

Abbreviations: OTU, operational taxonomic unit. Chao1, Shannon, and evenness were calculated using R software at the 2% distance level.

^*^ Total number of OTUs determined for the normalized sequence dataset

**Fig 2:**
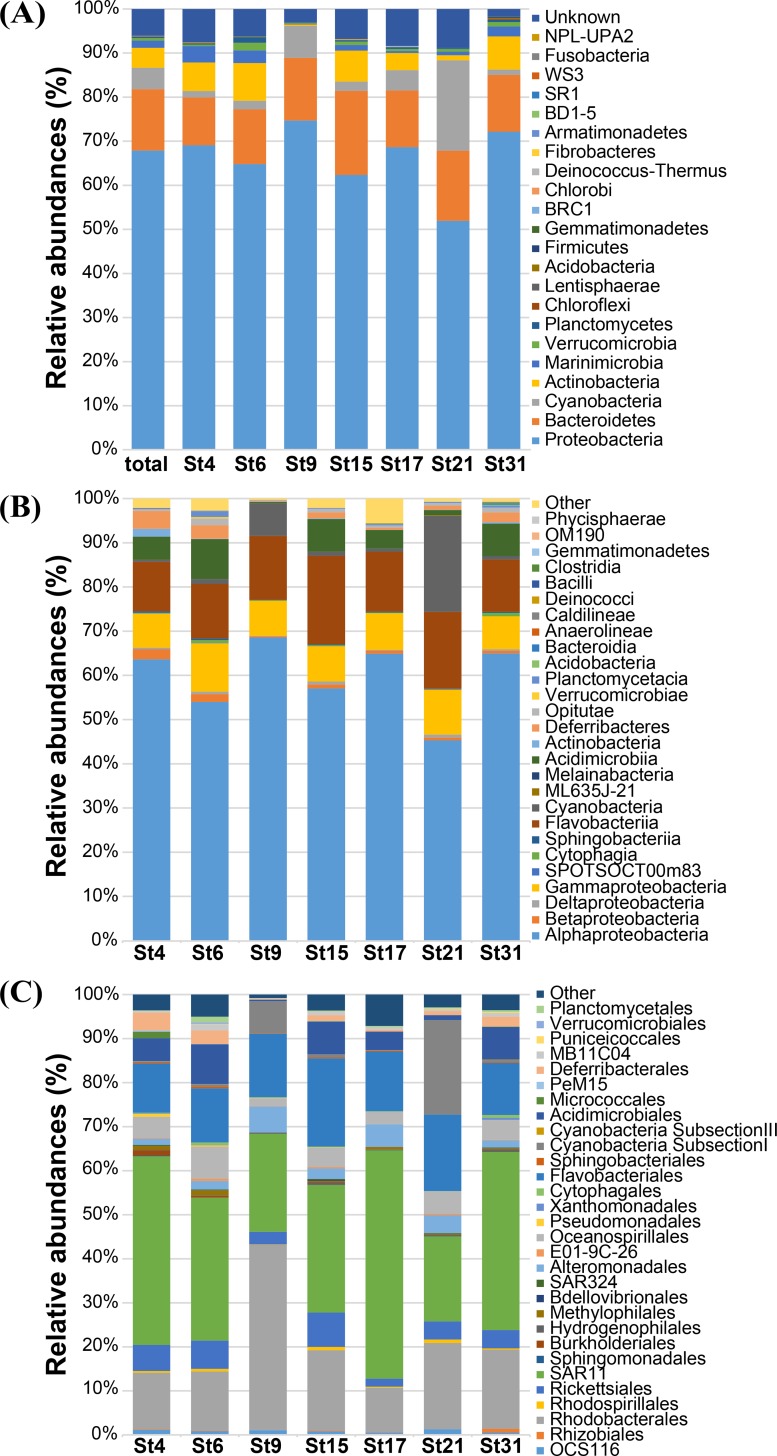
**Relative abundances of bacterial phylogenetic groups at (A) phylum, (B) class, and (C) order levels**.

The relative abundance of bacterial taxa was determined at different taxonomic levels from phylum to genus (S1 Table). A majority (94%) of the 19,860 bacterial sequences were assigned to 22 described bacterial phyla, but 6% of the sequences were affiliated with unknown phyla. Of 22 phyla, five phyla, *Proteobacteria*, *Cyanobacteria*, *Bacteroidetes*, *Actinobacteria*, and *Marinimicrobia* (SAR406 clade, misnamed *Deferribacteres* in SILVA), occupied 92.8% of the reads (Fig 2A). The phylum *Proteobacteria* was the predominant phylum in all samples, accounting for 67.9% of total bacterial sequences, followed by *Bacteroidetes*, *Cyanobacteria*, *Actinobacteria*, and *Marinimicrobia* (13.9%, 4.9%, 4.5%, and 1.6%, respectively). The other 17 phyla (*Verrucomicrobia*, *Planctomycetes*, *Chloroflexi*, *Lentisphaerae*, *Acidobacteria*, *Firmicutes*, *Gemmatimonadetes*, *Deinococcus*-*Thermus*, BRC1, *Chlorobi*, *Fibrobacteres*, SR1, WS3, *Armatimonadetes*, BD1-5, *Fusobacteria*, and NPL-UPA2) contributed to less than 1.0% of total bacterial abundance. Within the proteobacterial community, *Alphaproteobacteria* (58.2% of total sequences) and *Gammaproteobacteria* (7.9% of total sequences) were the predominant classes in all 7 samples (Fig 2B). The SAR11 clade and the order *Rhodobacterales* were the most prevalent groups in the *Alphaproteobacteria* (Fig 2C). The class *Flavobacteriia*, a member of the phylum *Bacteroidetes*, accounted for 13.5% of the total bacterial community and most of this class were found to be members of the order *Flavobacteriales* (Fig 2C). The bacterial community in the South Sea of Korea was mostly dominated by *Alphaproteobacteria*, *Bacteroidetes*, *Actinobacteria*, and *Cyanobacteria*, which was concurrent with the fact that they are major constituents in various marine systems [32, 33].

### Spatial distribution patterns of seawater bacterial communities

To investigate variations in bacterial assemblages among 7 sampling stations, as shown in Fig 2, differences in bacterial community structures were analyzed at phylum-, class-, and order-levels. Some phyla such as *Cyanobacteria* and *Actinobacteria* showed a clear difference in their abundance between the coastal and distal stations (Fig 2A). For example, much higher abundance of *Cyanobacteria* and lower abundance of *Actinobacteria* in distal stations (St9 and St21) were observed. The difference in bacterial community structures in the sampling stations was more evident in the order-level phylogenetic comparison (Fig 2C). To display distinct bacterial community structure between the coastal and distal stations, phylogenic groups comprising more than 1% of the total sequencing reads in at least one of the stations are summarized in Table 3.

**Table 3:** Comparison of relative abundance (%) of bacterial phylogenetic groups among the sampling stations.

Phylum/Class	Phylogenetic group	Group A		Group B			Group C	
		St4	St17	St6	St15	St31	St9	St21
***Alphaproteobacteria***	OCS116	1.1	0.5	0.8	0.8	0.5	1.0	1.2
	*Rhodobacterales*	11.9	9.2	12.6	17.0	17.6	40.8	17.9
	SAR116	5.4	1.6	5.8	7.1	3.6	2.7	3.7
	SAR11	39.6	47.5	30.4	27.0	39.8	21.5	17.6
	Other Alphaproteobacteria^*^	0.9	0.6	1.1	1.3	2.3	0.4	1.0
***Betaproteobacteria***	*Burkholderiales*	1.1	<0.1	0.4	0.2	0.2	<0.1	−
	OM43	0.8	0.5	1.0	0.2	0.2	<0.1	0.2
***Gammaproteobacteria***	*Alteromonadaceae*	0.9	2.2	1.7	1.5	1.4	5.5	3.6
	*Pseudoalteromonadaceae*	0.4	2.4	0.1	0.7	0.2	0.2	0.1
	SAR86	2.2	1.2	3.2	1.7	1.6	1.2	2.8
	ZD0405	1.1	1.0	2.1	1.5	1.7	0.4	0.9
	Other Gammaproteobacteria^*^	2.5	0.8	3.2	2.0	2.5	0.4	1.9
***Bacteroidetes***	*Cryomorphaceae*	2.4	1.8	1.6	2.8	2.0	1.2	1.5
	*Flavobacteriaceae*	7.3	10.0	8.3	14.4	8.3	11.9	12.9
	NS9	0.6	0.3	1.6	1.0	1.3	0.6	1.3
	Other Bacteroidetes^*^	0.5	0.4	0.9	0.4	1.3	0.2	0.1
***Cyanobacteria***	*Synechococcus*	0.1	0.1	0.5	0.7	0.4	7.0	19.1
	Unclassified *Cyanobacteria*	1.0	4.0	0.9	1.2	0.4	0.1	0.6
***Actinobacteria***	OM1	4.4	3.7	7.9	6.0	6.3	0.3	0.7
	Sva0996	0.2	−	0.5	0.8	1.0	−	0.3
	*Microbacteriaceae*	1.4	−	−	<0.1	0.1	−	−
***‘Marinimicrobia’***	SAR406	3.7	0.4	2.9	1.2	2.1	0.2	0.8
***Planctomycetes***	*Planctomycetaceae*	0.1	0.3	1.2	0.2	0.4	<0.1	0.2
***Verrucomicrobia***	MB11C04	0.3	0.4	1.4	0.7	0.8	0.2	0.4

Only phylogenic groups comprising more than 1% of the total pyrosequencing reads in at least one of the stations are shown. <0.1; less than 0.1%, −; not detected.

^*^ Sum of phylogenetic groups of which the relative abundance was less than 1% each.

**Fig 3:**
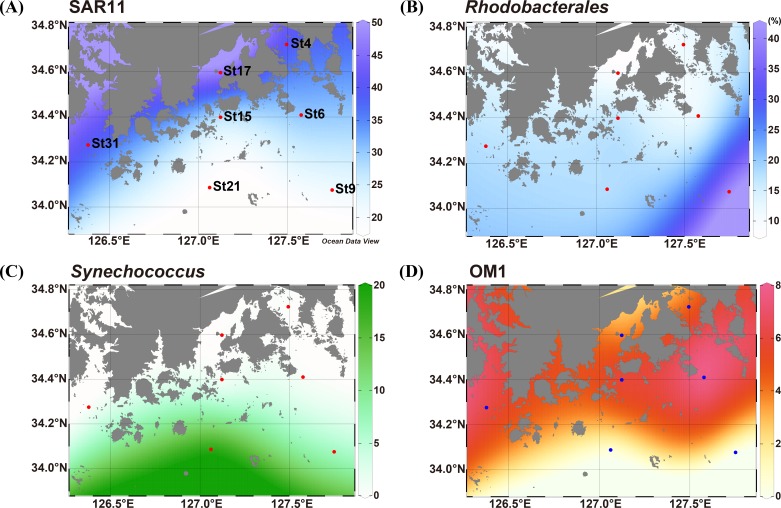
Gradient maps showing spatial distribution patterns of representative phylogenetic groups. (A) SAR11, (B) *Rhodobacterales*, (C) *Synechococcus*, (D) OM1. Numbers at color bars represent relative abundance (%) of each group in the bacterial community.

The phylum *Proteobacteria* was always predominant in all samples (>50% of total sequences at each station) but the relative abundance across the sampling stations was different. The proteobacterial abundance (52.0%) at St21 was much lower than that at other stations (62.3–74.7%), which might be affected by higher abundance of *Cyanobacteria* at a distal station, St21 (Fig 2). *Alphaproteobacteria* was the most dominant class in all stations and showed an apparent difference in the composition of phylogenetic groups among the stations at either order or family levels. Interestingly, two abundant groups of the class *Alphaproteobacteria*, the SAR11 clade (‘*Pelagibacterales*’) and the order *Rhodobacterales* showed a contrary distribution pattern along with distance from the coastline. The SAR11 clade was much more abundant in the stations closer to the coastline (St4 and St17; 39.6% and 47.5%, respectively) than in the stations far from the coast (St9 and St21; 21.6% and 17.6%, respectively). However, members of the order *Rhodobacterales* were more predominantly present in the distal stations (St9 and St21; 40.8% and 19.9%, respectively) than in the coastal stations (St4 and St17; 11.9% and 9.2%, respectively). The gradient distribution of relative abundance of SAR11 and *Rhodobacterales* is visualized on the map in Fig 3A and 3B. The distribution of the SAR11 clade was described by many studies and it has been revealed that the abundance of SAR11 is significantly high in euphotic marine surface waters and is correlated with dissolved organic matters produced by phytoplankton or chlorophyll *a* concentration [24, 30, 34]. Morris *et al*. observed that SAR11 surface clade (Ia) was positively correlated with phosphate concentration in the South Atlantic gyres [28, 30]. In the South Sea of Korea, DIN:DIP ratio in the coastal area was lower than the Redfield ratio (N:P = 16:1) and thus the coastal zone might be regarded as phosphorus-rich (4.96 in St4 and 10.44 in St17). By contrast, stations far from the coastline had much higher DIN:DIP ratio than the Redfield ratio, so that the oceanic area was considered to be phosphorous-limited (61.5 in St9 and 23.1 in St21). Our data showing the high abundance of SAR11 clade in relatively phosphate-rich coastal waters support these findings and suggest that the SAR11 clade actively responds to phosphate or phosphate-like dissolved organic matters produced by primary producers. In contrast to the SAR11 distribution, according to the study on the bacterial community dynamics in the Western English Channel, *Rhodobacterales* was prevalent in areas of lower nutrients but high primary productivity conditions [4]. The influx of freshwater into the South Sea of Korea, which leads to the change in nutrient composition, may cause an effect on the abundance of SAR11 in coastal regions while *Rhodobacterales* are more abundant in distal stations, which have relatively nutrient-poor conditions. *Betaproteobacteria* occupied minor fractions in all stations but the *Burkholderiales* and OM43 clade showed more abundance in the coastal stations (Table 3). Since the OM43 clade is a group of marine methylotrophs that are known to be present in productive coastal systems [35], the presence of the OM43 clade in the studied area represents a typical characteristic of coastal seawaters. In *Gammaproteobacteria*, members of the family *Alteromonadaceae* represented spatial variations, showing more abundance in the distal stations. The relative abundance of *Alteromonadaceae* was higher in St9 and St21 (5.5% and 3.6%, respectively) than in other coastal waters (0.9–2.2%). Bacterial community structure analyses at various biogeochemical conditions in the North Sea and in the north coast of Crete revealed that the habitat preferences of *Rhodobacterales* and *Alteromonadales* were in contrast to that of the SAR11 clade [36, 37]. Based on the sequence analyses of metatranscriptome and 16S rRNA gene, the SAR11 clade, *Rhodobacterales*, and gammaproteobacterial SAR92 clade were found to specifically react to different substrates in the marine ecosystem [38]. However, in some marine environments such as oligotrophic offshore waters in the South Adriatic Sea and the Atlantic Ocean, distribution of these major bacterial populations showed no apparent habitat preferences along the transect [39, 40]. In our study area, *Rhodobacterales*, *Alteromonadales*, and the SAR11 clade exhibited distinct spatial distribution, which might be attributed to competition for limited nutrients and various response to changing environmental conditions.

Most members of the phylum *Bacteroidetes* obtained from the seawater samples were assigned to the order *Flavobacteriales* (96.7% of *Bacteroidetes*), mainly composed of *Cryomorphaceae*, *Flavobacteriaceae*, and NS9 clade (Table 3). In the order *Flavobacteriales*, the bacterial community structure showed a strong station preference at the genus level (S2 Table). NS5 was the most prevalent group in *Flavobacteriales* and was present in a high proportion especially at costal stations St4, St6, St17, and St15 (39.7%, 34.6%, 36.8%, and 53.6% of *Flavobacteriales*, respectively). NS3a, NS4, and NS9 groups were also present abundantly in the coastal stations. However, *Formosa*, *Polaribacter*, and *Ulvibacter* were apparently prevalent in the distal stations (St9 and St21). Marine *Flavobacteriales* have been observed in a variety of marine environments, including coastal waters [41, 42], open ocean waters [40, 43], sediments [44], and hydrothermal vents [45]. As reported previously, our results suggest that different marine flavobacterial clades have distinct niches and different life strategies [29, 46], hence the differential occurrence of diverse flavobacterial groups reflects different environmental regimes in the studied area. The NS5 group has been known to respond accordingly to phytoplankton bloom since NS5 bacteria have enzymes for catalyzing phytoplankton-derived macromolecules, such as fucosidases [47]. An ecological study from the North Atlantic Ocean [29] also showed that members of the NS5 clade was significantly correlated with the abundance of nanophytoplankton. In this respect, the difference in flavobacterial community structures found among 7 seawater samples could be due to the influence of phytoplankton-derived organic matter.

Although *Cyanobacteria* and *Actinobacteria* occupied only 4.9% and 4.5% of the total bacterial community on average, respectively, they revealed distinctly different distribution patterns among the sampling stations (Fig 2). The abundance of *Cyanobacteria* exhibited an increasing trend along transects from coast to open ocean (Table 3) and they prevailed in the distal stations (7.3% at St9 and 20.5% at St21). Especially at St21, *Cyanobacteria* was more abundant than *Bacteroidetes* (15.9%), which was the second most abundant phylum in all other stations except St21. In other coastal stations, *Cyanobacteria* comprised less than 4.5% of sequencing reads at each station. Most of the cyanobacterial sequences recovered at St9 and St21 were affiliated with *Synechococcus* of which the proportion was 7.0% at St9 and 19.1% at St21 (Table 3). The gradient distribution of *Synechococcus* is visualized on the map in Fig 3C, which shows an increasing trend in their abundance along stations from the coastal to distal sea. The distribution of marine autotrophic picoprokaryotes *Synechococcus* and *Prochlorococcus* has been widely reported [48–50]. While the distribution of *Prochlorococcus* is limited in oligotrophic and tropical oceans between 40°S and 40°N, *Synechococcus* is known to be ubiquitously distributed in marine environments, specifically on the mesotrophic coastal sea surface. Studies on the picocyanobacterial abundance in the East China Sea including the South Sea of Korea and in the Yellow Sea showed that *Prochlorococcus* lineages were rare while *Synechococcus* occupied major fractions in the picocyanobacterial community in the waters of the continental shelf area [51, 52]. The prevalence of *Synechococcus* in the studied area coincided with this previous finding. The spatial and temporal studies in the Sargasso Sea presented that *Prochlorococcus* has a stronger requirement for inorganic nutrients than *Synechococcu*s [53]. Similarly, *Synechococcus* in the southern Mid-Atlantic Bight showed rapid growth during the summer season when macronutrients are limited [54]. The distal stations (St9 and St21) in the studied area were characterized by lower nutrient concentrations, which may favor the prevailing of *Synechococcus* in the distal stations. *Prochlorococcus* was not found in any of the sampling stations, which was considered to be related to the distribution characteristics of *Prochlorococcus*. In the Northwest Pacific, *Prochlorococcus* distribution in surface waters was found to be tightly associated with the main stream of the Kuroshio Current with high salinity, low nutrient content, and high temperature [51, 52]. The absence of *Prochlorococcus* at the sampling stations implies that the influence of the warm Kuroshio Current has not reached the study area.

The distribution of *Actinobacteria* across 7 stations showed a reverse pattern to that of *Cyanobacteria*. The proportions of *Actinobacteria* at costal stations (3.9–8.6%; St4, St6, St15, St17, St31) were much higher than those at distal stations (0.3–1.1%, St9 and St21). Most of *Actinobacteria* retrieved from the samples belonged to the OM1 clade (Table 3), an uncultured bacterial clade, that shows a distinct spatial distribution pattern on the gradient map (Fig 3D). It was reported that uncultured marine *Actinobacteria* were frequently recovered through 16S rRNA sequence data in various marine environments and showed higher abundance at coastal sites [28, 55]. Most of the coastal *Actinobacteria* sequences were identified to belong to OM1-related clusters and co-occurred with plentiful phytoplankton biomass in the South Atlantic Ocean [30]. In the South Sea of Korea, 4.5% of total 16S rRNA sequences accounted for *Actinobacteria* and the abundance in coastal stations was especially high compared to distal stations, St9 and St21, and most of them were affiliated with the OM1 clade, concurrent with the previous reports. OM1 was the most prevalent especially in St6 (7.9%) among 7 stations, and the concentration of chlorophyll *a* was also the highest in St6.

**Fig 4:**
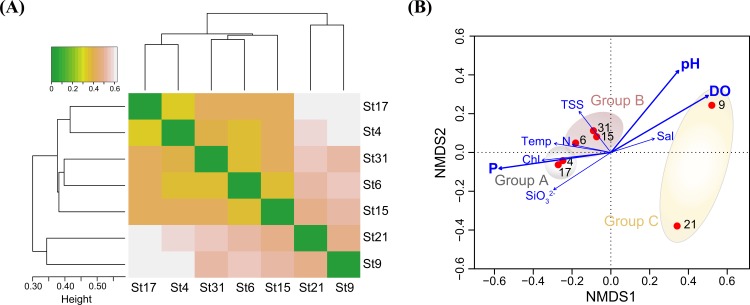
Clustering analysis based on OTUs of 7 seawater samples. (A) Dendrogram and heat map based on Bray-Curtis distances showing the clustering of bacterial community of sampling stations, (B) A non-metric multidimensional scaling plot showing the relationships among sampling stations. Correlations between environmental variables and the first two dimension axes are represented by the arrows, and variables showing significant correlation (p < 0.05) are indicated in bold. Temp, temperature; Sal, salinity; DO, dissolved oxygen; TSS, total suspended solids; P, phosphate; N, dissolved inorganic nitrogen; Chl, chlorophyll *a*.

To better understand bacterial distribution patterns with respect to spatial and environmental variables, pyrosequencing data were statistically analyzed. With the normalized OTU data by random subsampling, a cluster analysis, using Bray-Curtis distance dissimilarity and weighted pair group average linkage, was performed in order to investigate differences in the bacterial community composition along the sampling stations. The clustering analyses showed that bacterial communities in the South Sea of Korea could be distinctly separated into two groups (Fig 4A). The first group consists of bacterial communities from the stations of coastal waters (St17, St4, St31, St6, St15) and the second group included communities of distal stations (St9 and St21; 60–90 km apart from the coast). The coastal group was further separated into two groups: bacterial communities collected at stations near the coastline (St4 and St17; 10–20 km apart from the coast) and those from stations of intermediated area (St6, St15, and St31; 30–50 km apart from the coast). As mentioned above, this statistical clustering pattern was consistent with arbitrary grouping based on distances from the coastlines, indicating that bacterial communities in the South Sea of Korea were influenced by spatial distribution from the coastline and corresponding trophic status of water masses. This clustering pattern among the bacterial communities at 7 different stations was also confirmed by Chord distance-based non-metric multidimensional scaling (NMDS). The NMDS plot also revealed a primary clustering by microbial habitats (Fig 4B). Similar to the clustering analysis result, bacterial communities at St4 and St17 (coastal, Group A), St6, St15, and St31 (intermediate, Group B), and St9 and St21 (distal, Group C) could be separately grouped on the plot. The analysis of similarities (ANOSIM) showed that separation into 3 groups based on the cluster analysis and NMDS were well supported statistically (R = 0.8, p < 0.001). Correlation analyses between bacterial communities and environmental variables showed that PO_4_^3−^ (p < 0.01), dissolved oxygen (p < 0.01), and pH (p < 0.05) were strongly correlated with bacterial assemblages. However, other parameters such as temperature, salinity, chlorophyll *a*, DIN, and TSS did not show significant correlations (p > 0.1).

**Fig 5:**
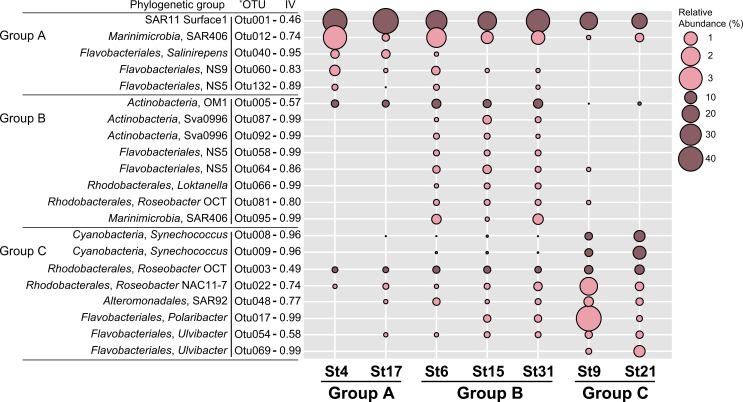
Bubble plots showing the relative abundance of indicator OTUs in each of the 3 groups of the sampling stations. The size of the bubble indicates the relative abundances (%) of each OTU in each of the 7 sampling stations. Indicator values are displayed next to each OTU. Consensus taxonomic groups of each indicator OTU are presented on the left side of bubble plots. ^*^ OTUs with IV > 0.4 and p < 0.05.

IndVal analysis was additionally performed to identify indicator OTUs that represent characteristics of the bacterial community in each of the 3 groups of sampling stations and the results showing the relative abundance of indicator OTUs are presented in Fig 5 as bubble plots. The abundance bubble plots showed that several indicator OTUs dominated the specific group of sampling stations and represented a similar distribution pattern within each group. For example, OTU001 (of SAR11), OTU040 (of *Salinirepens*), and OTU060 (of NS9) in Group A (coastal stations), OTU005 (of OM1), OTU087 (of Sva0996), OTU058 (of NS5), and OTU066 (of *Loktanella*) in Group B (intermediate stations), and OTU022 (of NAC11-7), OTU008 (of *Synechococcus*), OTU048 (of SAR92), and OTU017 (of *Polaribacter*) in Group C (distal stations) were representative indicator OTUs in each group of sampling stations. The representative 16S rRNA gene sequences of OTU001 showed 100% similarity with strain HTCC7211, a member of the SAR11 Ia.3 subclade. Metagenome analysis revealed that the SAR11 Ia.3 subclade is widely distributed at the surface of the oceans worldwide such as the Sargasso Sea [24], where strain HTCC7211 was isolated, and the subclade is known to be highly correlated with warm temperature [56]. The relatively warm temperature in the coastal stations (Group A) could be one of the factors affecting the spatial distribution of the SAR11 clade in the South Sea of Korea. In contrast to the SAR11 clade, OTU066, OTU081 and OTU003, and OTU022 affiliated to the *Loktanella*, OCT, and NAC-11 clades, respectively, belonging to *Rhodobacterales*, were analyzed to represent indicator OTUs for Group B and Group C. The OM1 and Sva0996 clades belonging to uncultured marine *Actinobacteria* clades were found to thrive in high nitrate concentration and high primary productivity-showing area in the Sargasso Sea [57] and the Columbia River coastal margin [58]. In our study area, OTU005 of the OM1 clade and OTU087 and OTU092 of the Sva0996 clade were good indicator OTUs for coastal stations, especially for intermediate stations.

OTU012 in Group A and OTU095 in Group B belonged to the SAR406 clade and OTU132 in Group A and OTU058 and OTU064 in Group B belonged to the NS5 clade, indicating specific members of the SAR406 and NS5 clades were good indicators for coastal waters (Group A and Group B). As shown in the clustering dendrogram (Fig 4), since bacterial communities in the South Sea of Korea were separated into the coastal lineage (Group A and B) and the distal lineage (group C), different OTUs in the same phylogenetic group could be indicator taxa for both Group A and Group B. Similarly, OTUs in the *Roseobacter* clade (OTU003, OTU022), OTUs in *Synechococcus* (OTU008, OTU009), and OTUs in the *Flavobacteriaceae* (OTU017, OTU054, OTU069) were good indicators for distal sampling stations (Group C). The representative 16S rRNA gene sequences of OTU008 and OTU009 were 100% identical to those of *Synechococcus* strains WH8016 and CC9902, respectively. Strains WH8016 and CC9902 belongs to the subclades Ⅰ and Ⅳ of *Synechococcus* subcluster 5.1 that is the most abundant *Synechococcus* cluster throughout the ocean. Clades Ⅰ and Ⅳ co-occurred in the Arctic Ocean, the Atlantic Ocean, and off the Californian coast, but have been rarely found in the Red Sea, the Arabian Sea, and costal boundary area of the Indian Ocean [49, 59, 60], suggesting their confined distribution in higher latitudes (above 30 °N and below 30 °S). Interestingly, the indicator OTUs belonging to *Flavobacteriales* were present in all groups A, B, and C, but the indicators of the coastal region (Group A and B) and the oceanic region (Group C) were clearly distinct (Fig 5). OTUs of the NS9 and NS5 clades were indicators for the coastal stations, while OTUs of *Polaribacter* and *Ulvibacter* were indicators for the distal stations. OTU060 of the NS9 clade formed a monophyletic cluster with the genus *Fluviicola*, reported as a prevalent flavobacterial group near the mouth of the Peal estuary [61]. The NS groups including NS5 and NS9 were reported to be the most abundant flavobacterial groups retrieved from coastal North Sea and bacterial sequences affiliated to the genera *Polaribacter* and *Ulvibacter* have been widely discovered from pelagic marine environment [62], which shows a similar pattern of distribution found in this study area of the South Sea of Korea. Comprehensively, statistically significant indicators of each clustering group listed in Fig 5 corresponded to the dominant taxonomic groups in the sampling stations and reflected spatial distribution of the bacterial community in the South Sea of Korea.

## Conclusion

In this study, we analyzed 16S rRNA gene amplicon sequences obtained from 7 different stations to characterize changes in the marine bacterial community structure that reflect apparent responses to spatial heterogeneity of environmental factors. The 16S rRNA gene abundance of diverse phylogenetic groups in the sampling stations revealed that specific niches were formed according to the distances from coastlines, which led to the prevalence of specific dominant taxonomic groups. Although a restricted number of samples caused limitations in deriving conclusions on bacterial distribution reflecting microbial endemism, there was an obvious taxa distribution pattern both within and between the sampling stations in the South Sea of Korea. Bacterial assemblages at coastal stations were highly dominated by the SAR11 clade, actinobacterial OM1 clade, and NS5 and NS9 groups in the order *Flavobacteriales*. In contrast, members of *Rhodobacterales*, *Synechococcus*, *Polaribacter*, and *Ulvibacter* were good indicators for representing the bacterial community of distal stations. These bacterial community structures in the South Sea of Korea showed statistically significant relationships with spatial characteristics of each station and environmental factors such as phosphate. Unfortunately, many indicator taxa identified in this study, belonging to the SAR406, OM1, NS5, and NS9 clades, are as-yet-uncultured. Cultivation of these uncultured groups of bacteria would reveal their physiology in the diverse marine environmental regimes, which will show their spatiotemporal adaptation strategies in the ocean.

## Supporting information

S1 Table Summarized information on pyrosequencing data and taxonomic assignment. (DOCX)

S2 Table Relative abundance (%) of members of the order *Flavobacteriales*, represented as percentage of total *Flavobacteriales*. (DOCX)
