# Supplementary material for: Characterization of spatial distribution of the bacterial community in the South Sea of Korea
Source: PLoS One. 2017 Mar 17;12(3):e0174159. doi: 10.1371/journal.pone.0174159 (PMC5357018; doi:10.1371/journal.pone.0174159)
Supplement: S1 Table — (DOCX) [file pone.0174159.s001.docx]

**Supporting Information**

|  | St4 | St6 | St9 | St15 | St17 | St21 | St31 | Total |
| --- | --- | --- | --- | --- | --- | --- | --- | --- |
| No. of total sequences | 7481 | 4130 | 6015 | 5733 | 3935 | 2228 | 1511 | 31033 |
| No. of bacterial sequences | 4686 | 1974 | 5157 | 3134 | 2519 | 1438 | 952 | 19860 |
| No. of OTUs^*^ | 540 | 330 | 486 | 484 | 373 | 232 | 232 | 1714* |
| No. of phyla | 18 | 12 | 10 | 13 | 14 | 9 | 12 | 22 |
| No. of classes | 36 | 22 | 20 | 30 | 28 | 18 | 24 | 55 |
| No. of orders | 69 | 53 | 43 | 63 | 50 | 40 | 19 | 116 |
| No. of families | 104 | 80 | 64 | 95 | 70 | 55 | 78 | 187 |
| No. of genera | 167 | 111 | 109 | 147 | 103 | 84 | 114 | 315 |

**S1 Table. Summarized information on pyrosequencing data and taxonomic assignment.**

^*^ Total number of OTUs determined for the entire dataset
