# Supplementary material for: Characterization of spatial distribution of the bacterial community in the South Sea of Korea
Source: PLoS One. 2017 Mar 17;12(3):e0174159. doi: 10.1371/journal.pone.0174159 (PMC5357018; doi:10.1371/journal.pone.0174159)
Supplement: S2 Table — (DOCX) [file pone.0174159.s002.docx]

**Supporting Information**

**S2 Table. Relative abundance (%) of members of the order *Flavobacteriales,* represented as percentage of total *Flavobacteriales.***

| Order | Genus/clade | Group A | | Group B | | | Group C | |
| --- | --- | --- | --- | --- | --- | --- | --- | --- |
|  |  | St4 | St17 | St6 | St15 | St31 | St9 | St21 |
| *Flavobacteriales* | *Fluviicola* | 3.3 | 3.7 | 4.5 | 5.9 | 9.8 | 2.7 | 6.1 |
|  | *Owenweeksia* | 9.8 | 5.0 | 6.5 | 7.7 | 4.1 | 5.0 | 2.2 |
|  | *Salinirepens* | 7.3 | 3.7 | 1.6 | 1.0 | 0.0 | 0.0 | 0.4 |
|  | *Aequorivita* | 1.6 | 0.3 | 0.0 | 0.2 | 0.0 | 0.0 | 0.0 |
|  | *Algibacter* | 0.0 | 0.0 | 0.0 | 0.2 | 0.8 | 0.1 | 0.0 |
|  | *Formosa* | 1.0 | 3.4 | 0.8 | 7.9 | 7.3 | 20.5 | 14.8 |
|  | *Polaribacter* | 5.1 | 3.7 | 5.7 | 8.7 | 9.8 | 29.5 | 12.2 |
|  | *Psychroserpens* | 0.0 | 0.0 | 0.0 | 0.0 | 0.8 | 0.5 | 0.4 |
|  | *Ulvibacter* | 0.8 | 0.6 | 0.8 | 1.5 | 4.1 | 4.6 | 5.7 |
|  | *NS2b* | 1.4 | 2.8 | 5.3 | 7.0 | 4.9 | 3.7 | 14.4 |
|  | *NS3a* | 1.8 | 0.0 | 0.0 | 0.5 | 0.0 | 0.0 | 0.0 |
|  | *NS4* | 14.5 | 11.5 | 16.7 | 10.7 | 11.4 | 8.3 | 8.7 |
|  | *NS5* | 39.7 | 53.6 | 34.6 | 36.8 | 22.0 | 14.2 | 23.6 |
|  | *NS7^*^* | 0.6 | 2.2 | 0.8 | 2.5 | 0.8 | 1.9 | 1.3 |
|  | *NS9^*^* | 5.1 | 2.5 | 13.0 | 5.2 | 9.8 | 4.1 | 7.9 |
|  | NS10 | 0.0 | 0.0 | 0.0 | 0.2 | 0.0 | 0.7 | 0.4 |

^*^ According to SILVA taxonomy, NS7 and NS9 clades represent family-level taxa.
